# Neurogenesis as a Tool for Spinal Cord Injury

**DOI:** 10.3390/ijms23073728

**Published:** 2022-03-28

**Authors:** Katerina Havelikova, Barbora Smejkalova, Pavla Jendelova

**Affiliations:** 1 Institute of Experimental Medicine, Czech Academy of Sciences, Vídeňská 1083, 14220 Prague, Czech Republic; katerina.neumannova@iem.cas.cz (K.H.); barbora.smejkalova@iem.cas.cz (B.S.); 2 Department of Neuroscience, Second Faculty of Medicine, Charles University, 15006 Prague, Czech Republic

**Keywords:** neurogenesis, spinal cord injury, ependymal stem cells, astrocytes, reprogramming, spinal canal, physical factors, valproic acid, growth factors, neuroinflammation

## Abstract

Spinal cord injury is a devastating medical condition with no effective treatment. One approach to SCI treatment may be provided by stem cells (SCs). Studies have mainly focused on the transplantation of exogenous SCs, but the induction of endogenous SCs has also been considered as an alternative. While the differentiation potential of neural stem cells in the brain neurogenic regions has been known for decades, there are ongoing debates regarding the multipotent differentiation potential of the ependymal cells of the central canal in the spinal cord (SCECs). Following spinal cord insult, SCECs start to proliferate and differentiate mostly into astrocytes and partly into oligodendrocytes, but not into neurons. However, there are several approaches concerning how to increase neurogenesis in the injured spinal cord, which are discussed in this review. The potential treatment approaches include drug administration, the reduction of neuroinflammation, neuromodulation with physical factors and in vivo reprogramming.

## 1. Introduction

A spinal cord injury (SCI) is an insult to the spinal cord resulting in a change, either temporary or permanent, in the cord’s normal motor, sensory or autonomic function. Patients with SCI usually have permanent and often devastating neurologic deficits and disability. The major consequences are tissue damage, the death of neurons and disruption of neuronal connections; all lead to the loss of mobility, sensation or autonomic function. The pathophysiology of traumatic SCI has two phases: primary and secondary injuries. Primary injury is the result of trauma in which spinal cord tissue is violently damaged. In this phase of injury, within a few minutes, cell death and damage to the vasculature and blood–spine barrier occurs [1]. Simultaneously, a cascade of events is initiated that leads to extensive secondary damage [2]. First, inflammation and hemorrhage develop in the tissue, leading to necrosis and ischemia [3]. Massive collections of inflammatory cells appear at the injury site within 12–24 h, where the first inflammatory cells are neutrophils, followed by lymphocytes and then macrophages [2]. Damage in the tissue continues 2–4 days after injury with the disruption of ion homeostasis [4], glutamate excitotoxicity [5], production of reactive oxygen species [6], lipid peroxidation [7], impaired autophagy [8], accumulation of nitric oxide flux [9], glial scar formation [10] and energy failure [11]. In the months and years after injury, the subacute phase transitions to a chronic phase in which central cavitation occurs, glial scar formation continues and changes in ion channels and receptors occur. Oligodendrocyte apoptosis leads to demyelination and damage to the surviving axons [12]. Regeneration in the form of sprouting axons [13] is prevented by the non-permissive environment formed by extracellular matrix molecules during the subacute and chronic phases of injury. The glial scar formed by reactive astrocytes around cystic cavities prevents damage to the adjacent tissue but, at the same time, together with extracellular matrix proteins, such as chondroitin sulfate proteoglycans, tenascin and NG2 proteoglycan, limits axon regeneration and plasticity [14].

To date, there is no effective treatment for SCI. One possible approach to SCI treatment could be provided by stem cells (SCs). Studies have mainly focused on the transplantation of exogenous SCs, but the induction of endogenous SCs has also been considered as an alternative. This approach would avoid the risks accompanying exogenous SC transplantations, such as immunoreactivity or the formation of tumors.

## 2. Endogenous Neural Stem Cells

Currently, there are two well-described main regions in the mammalian brain that contain neural stem cells (NSCs): the subgranular zone in the dentate gyrus and the subventricular zone of the lateral ventricles. Cells from the subventricular zone generate doublecortin positive neuroblasts, which migrate to the olfactory bulb and differentiate into olfactory neurons, tuning the fine plasticity of the olfactory system. The subgranular zone of the gyrus dentate is involved in learning and memory (reviewed in [15]). Thereafter, the striatum, along with other areas, were reported as an additional neurogenic niche in humans [16,17]. While the differentiation potential of neural stem cells in the brain neurogenic regions has been known for decades, there are ongoing debates regarding the multipotent differentiation potential of the ependymal cells of the central canal in the spinal cord (SCECs). SCECs form a continuous epithelial sheet lining the ventricles and the central canal of the spinal cord. These cells are of glial lineage but have many epithelial characteristics, including a basement membrane, cell–cell junctions and motile cilia [18].

The first reports regarding the renewal of ependymal cells in a mouse spinal cord appeared in 1962 using radioactivity experiments [19]. The authors reported newly dividing cells in SCI, which were identified as astrocytes and oligodendrocytes but not neurons. These findings were later confirmed by several authors supporting the statement that neurogenesis is not present in the rodent spinal cord [18,20,21]. New isolation, expansion and culturing of cells in the form of neurospheres brought new evidence that cells from the spinal cord can not only self-renew but can form neurospheres and generate in vitro neuronal cells [22]. Subsequently, it was necessary to identify these multipotent stem-cell-like cells. There were several possible candidates: astrocyte precursor cells, oligodendrocytes precursor cells and ependymal cells. Based on genetic fate mapping, it was shown that while oligodendrocyte progenitors can self-renew and give rise to new mature oligodendrocytes, only ependymal cells are multipotent and neural stem cell activity in the intact and injured adult mouse spinal cord is restricted to this cell population [18,21]. In contrast, oligodendrocyte progenitor cells expressing markers such as nerve/glial antigen-2 (NG2) and/or platelet-derived growth factor receptor alpha (PDGFRα), also known as NG2-glia, NG2-cells or polydendrocytes, are scattered in the white matter and gray matter throughout the central nervous system and represent the main proliferating cell population in the intact spinal cord; however, they do not display in vitro neural stem cell properties [18].

The discovery of NSCs in the spinal cord was prolonged due to their lack of activity under physiological conditions. SCECs can be activated during pathological conditions, such as SCI, inflammation or neurodegeneration. Spinal cord ependymal cells start to proliferate, migrate to the site of damage and differentiate. The phenotype of the differentiated cell depends on the surrounding environment. For example, in the model of multiple sclerosis as an inflammatory disease, SCECs differentiate into oligodendrocytes and possibly into neurons [23]. In a neurodegenerative disease, such as amyotrophic lateral sclerosis, SCECs mostly differentiate into astrocytes [24]. SCECs can also be activated by physical activity; proliferating nestin-positive cells were detected 4–7 days after running wheel training in rats [25].

### SCECs in Spinal Cord Injury

**Figure 1:**
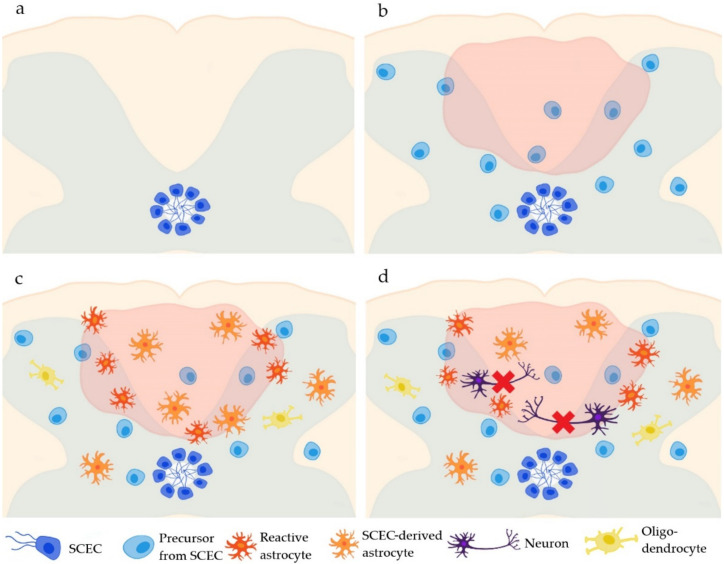
The fate of the ependymal cells of the central canal in the spinal cord (SCECs). Under physiological conditions, SCECs border the central canal of the spinal cord (**a**). After an SCI, SCECs start to proliferate (**b**) and differentiate mostly into astrocytes (2 weeks post-injury) and partly into oligodendrocytes (4 months post-injury) (**c**), but not into neurons (**d**).

Following spinal cord insult, SCECs start to proliferate and differentiate mostly into astrocytes and partly into oligodendrocytes, but not into neurons [18,21,25,26,27] (Figure 1). A glial scar has both beneficial and detrimental effects on recovery after spinal cord injury [28,29]. Therefore, the differentiation of SCECs to astrocytes plays a substantial role in SCI healing. It was shown that astrocytes originating from SCECs help to form a glial scar bordering the SCI [18]. Without these astrocytes, the damage would spread to the surrounding tissue and result in an enlarged lesion volume, neuronal degeneration and a worse functional outcome [30].

A glial scar is, however, not only formed from the astrocytes derived from SCECs but also from astrocytes derived from astrocyte progenitors. These two populations contain different properties. A few days following an SCI, the progenitor cells from the central canal region migrate in the direction of the injury center and downregulate ependymal cell markers, such as Sox2, Sox3 and FoxJ1. These cells further divide and differentiate into astrocytes, which form the core of a glial scar, whereas astrocytes from astrocyte progenitors migrate toward the periphery of the glial scar [18]. In addition, astrocytes from SCECs do not usually express GFAP, but they produce laminin, which helps axons to grow, whereas GFAP+ astrocytes from dividing astrocyte progenitors produce chondroitin sulfate proteoglycans (CSPGs) that inhibit the axon growth [21]. SCEC progenitors also exert a neurotrophic effect that is required for the survival of the surrounding neurons [30], as the progenitors express several growth factors and increase expression after differentiation when expanded in vitro [31].

**Figure 2:**
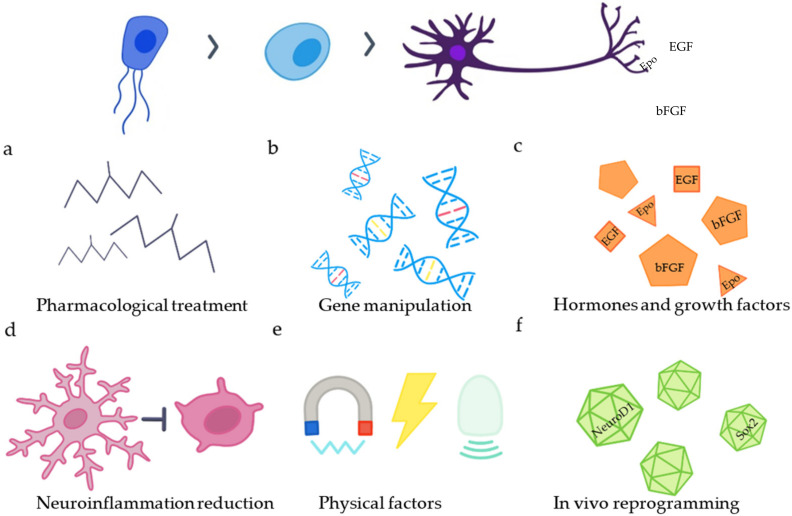
Strategies to increase endogenous neurogenesis. The differentiation of SCECs into neurons can be enhanced by (**a**) application of different drugs (VPA, RA, Ro3303544), (**b**) manipulation of genes (BAF45D peptide, connexin 50), (**c**) application of hormones or growth factors (EGF, bFGF, Epo, substance P), (**d**) reduction in neuroinflammation, (**e**) application of physical factors and (**f**) in vivo reprogramming of reactive astrocytes and NG2 glia.

Unfortunately, the beneficial effect of SCEC-differentiated astrocytes is not sufficient for spinal cord regeneration. For full recovery from an SCI, there is a need to replace destroyed neurons and also oligodendrocytes to myelinate them. Therefore, the activation of endogenous NSCs and their neuronal differentiation induction would be an interesting approach to generate the lost population of neurons. The first studies, which focused on overcoming the gliogenic environment in the spinal cord, were not successful. Spinal cord neural stem cells genetically modified to express neurogenin 2 (Ngn2) differentiated into neurons in vitro, but not after transplantation into an SCI [32]. In contrast, other studies have confirmed the ability of neural stem cells that expanded from the adult spinal cord to differentiate into neurons in the neurogenic niche in the dentate gyrus [33]. Therefore, different strategies to facilitate neuronal differentiation from SCEC have started to emerge (Figure 2).

## 3. Approaches to Promote Neurogenesis

### 3.1. The Differentiation of SCECs into Neurons

A small environmental change can induce NSCs to produce neurons instead of glial cells. The addition of valproic acid (VPA) to embryonic brain NSCs transplanted into the spinal cord led to the production of neurons instead of only astrocytes. These differentiated neurons were able to connect into the existing network and form synapses with endogenous neurons, which led to functional improvement in mice [34].

A similar effect was described for the endogenous SCECs. In vitro experiments clearly indicated that SCECs have an intrinsic capacity of producing neurons and their fate depends on the surrounding environment. SCECs cultured with VPA have increased neuronal induction and promote neuronal differentiation, while astrocytic differentiation is suppressed. Cell cycle regulator p21(Cip/WAF1) and proneural genes *Ngn2* and *NeuroD1* were increased in these two processes, respectively [35,36]. VPA has many pharmacological effects and is already used as a medicament to treat epilepsy and bipolar disorder. In rat SCI, delayed treatment with VPA led to in vivo increased neurogenesis; the evidence of which was based on the newborn neuron marker doublecortin and the mature neuron marker neuron-specific nuclear protein, which were enhanced in the epicenter of the SCI and neighboring tissue [36]. VPA-induced HDAC inhibition led to the activation of the Wnt/β-catenin pathway and, consequently, decreased GSK-3 activity. Reduced GSK-3 activity results in the upregulation of the substrate of GSK-3-cytoplasmic levels of the transcription factor β-catenin, that is, they were negatively regulated through phosphorylation-dependent degradation [37,38]. Moreover, neurogenesis induced using VPA can create a more protective environment in the tissue due to the expression of neurotrophic factor BDNF in newborn neurons. The upregulation of BDNF via BNDF-Trkb, leads to the subsequent activation of MAPK/Erk pathways [39,40]. The increase in Erk indirectly inhibits GSK-3; therefore, neuroprotection and neurogenesis are coupled in the VPA treatment (reviewed in [41]).

The inhibition of GSK-3 appears to be a potential target for increased neurogenesis, as shown in further studies. A recent study showed that the application of GSK-3 inhibitor Ro3303544 on SCECs isolated from mouse spinal cords and leads to the increased expression of early neuronal marker βIII-tubulin and late neuronal marker MAP2. Similarly, in mouse SCI, treatment with GSK-3 inhibitor Ro3303544 not only increased the survival of neurons but also more newborn neurons formed synapses close to the injury epicenter. This all led to improved motor recovery and decreased astrogliosis in the injury epicenter [42].

SCEC neuronal differentiation can be enhanced by substance P. This neuropeptide is involved in the synthesis of growth factors and cytokines and, therefore, in cell proliferation. The injection of substance P leads to functional improvement by activating SCECs. SCEC activation increases their proliferation and differentiation into neurons and decreases proliferation and differentiation into astrocytes in vivo. In vitro, it enhances neuronal differentiation by activating the Erk1/2 pathway [43]. These findings are consistent with previous studies with VPA and Ro3303544 since Erk1/2 inhibits GSK3 and vice versa [44,45,46].

VPA also synergizes with all-trans retinoic acid (RA), an important regulator during embryonic development, when it defines the anterior/posterior axis. RA increases the neurogenesis of SCECs in vitro and reduces their differentiation into astrocytes [35,47]. RA can be combined with the growth factors bFGF/EGF, which promote axon growth and improve the neuronal differentiation of the embryonic brain NSCs [48]. Neural differentiation induced by RA can be disrupted by knocking down the BAF45D protein [49]. This protein is present in the developing mouse cortex and the adult mouse hippocampus. Silencing its expression leads to the inhibited expression of Pax6, which is a neurogenic transcription factor contributing to neurogenesis. In the spinal cord, BAF45D is expressed in SCECs, neurons and oligodendrocytes, but not in astrocytes. After SCI, the expression of BAF45D in SCECs is decreased, and thus, proliferating SCECs mainly differentiate into astrocytes instead of neurons [50]. Therefore, the targeting of this peptide may be another potential way to affect neurogenesis after an SCI.

The important molecules that are also known from the developmental stage are connexins, which are proteins that form gap junctions. These molecules play a role not only in development but also in proliferation and differentiation [51,52]. The expression of Sox2, which is a neural progenitor marker participating in the conversion of endogenous glia into neurons [53], is regulated by connexin 50. Silencing connexin 50 resulted in the downregulation of connexin 50, while its overexpression led to more Sox2 cells in the SCEC population [52]. A subpopulation of SCECs lateral from the central canal is connected with gap junctions. This connection is downregulated at the end of development but upregulated again after an SCI. This increase in the gap junction coupling correlates with connexin 26 upregulation and leads to the recovery of SCEC proliferation. On the other hand, the blocking of this connexin decreases SCEC proliferation [51]. Therefore, connexins and gap junctions are important for SCEC proliferation and, thereby, a potential target.

Erythropoietin (Epo) is a hormone that is important for erythropoiesis. In spinal cord injury treatment, Epo signaling is involved in several neuroprotective processes, such as anti-apoptotic and anti-inflammatory functions and edema reduction [54,55]. Meanwhile, in the brain neurogenic regions, Epo application increases the number of newly generated neurons [56] in the healthy spinal cord and does not affect SCEC proliferation. However, after SCI, Epo treatment significantly promotes SCEC differentiation into neurons and oligodendrocytes [57].

Growth factors (GFs) are a family of proteins that are involved in the regulation of development and function, the survival of neurons, neurotransmitter release, recovery of synaptic function and axon regeneration [58]. However, various types of growth factors have different functions regarding repairing SCI [59]. Therefore, it is apparent that the use of growth factors is one of the most tested approaches to promote neurogenesis. The epidermal growth factor (EGF) and basic fibroblast growth factor (bFGF, also known as FGF2) were shown to be able to jointly, but not alone, induce the proliferation of SCECs in vitro [22,60]. The same was even shown later in vivo [61,62], where the infusion of either EGF or bFGF alone into the spinal cord had no effect, but their combination led to the increased proliferation of SCECs. The same authors later reported that the infusion of EGF + bFGF into the injured spinal cord of rats led to the proliferation and migration of SCECs to the site of injury and functional improvement. However, the mechanism responsible for functional improvement remained unclear, as there were no new neurons or oligodendrocytes detected [63]. The effect of growth factors may remain behind the mechanisms for increased proliferation of SCECs after exercise, as physical activity increases the expression of GFs [25].

The effect of GFs can be facilitated by genetic manipulation. The combination of GF with transcription factor overexpression can support neurogenesis and/or oligodendrogliogenesis. The transcription factor Ngn2 induced neuronal differentiation in vitro but had almost no effect in vivo. However, in combination with the previously mentioned EGF and FGF2 (bFGF), the retroviral-induced overexpression of Ngn2 led to the production of new neurons in vivo [64]. The same viral vector was used to overexpress Mash1, which resulted in the production of new oligodendrocytes in the same experiment [64]. Due to the short half-life of GFs and the need for their sustained release, GFs are often delivered in combination with biomaterials. For example, a biodegradable chitosan scaffold was loaded with NT3 and inserted into a 5 mm gap in a completely transected spinal cord. Slowly released NT3 activated SCECs to migrate into the lesion area and differentiate into neurons, which formed a functional network that led to the functional recovery of rats with an SCI [65,66]. As this approach was successful it was also repeated in monkeys with a hemisection model of SCI, where it led to neuroregeneration, the growth of cortico-spinal tract (CST) axons through the lesion and functional recovery [67]. A sodium hyaluronate scaffold was combined with a ciliary neurotrophic factor [68]. In this study, a scaffold with neurotrophic factor was implanted into a 5 mm gap after the removal of a 5 mm T8 segment of a spinal cord in rats. The scaffold releasing ciliary neurotrophic factor (CNTF) led to the activation of endogenous SCECs, their migration to the injury site, differentiation into neurons and even the formation of functional synapses, followed by the improvement of motor and sensory functions [68]. Injectable hydrogels can fill the lesion cavities and, therefore, appropriately integrate into the tissue and modify the environment for better regeneration [69,70]. Hydrogels can support pro-regenerative macrophage polarization and angiogenesis, leading to axonal regeneration and neurogenesis. The injection of a functional ECM-resembling, self-assembling, peptide nanofiber hydrogel with CNTF, BDNF and NGF growth factors has led to the axonal regeneration, myelination, proliferation and neuronal differentiation of SCECs that connect to the CST, which resulted in the recovery of locomotion in rats with a SCI [70].

In addition to the direct administration of GFs, GF receptors are important targets. Myelin-associated inhibitors (MAIs) and chondroitin sulfate proteoglycans (CSPGs) are major components of the inhibitory microenvironment following an SCI. The activation of EGF receptor (EGFR) by MAIs suppresses the neuronal differentiation of NSCs [71,72]. Therefore, blocking this signaling pathway might lead to increased neuronal differentiation. This was demonstrated in studies using an EGFR antibody, such as the drug cetuximab, which is usually used for cancer treatment [73,74,75,76]. Cetuximab released from the collagen scaffold increased neuronal and decreased astrocyte differentiation in vitro [75]. Moreover, the transplantation of this scaffold into SCI induced neuronal differentiation, reduced astrogliosis and improved axonal regeneration in vivo [74,75,76]. Instead of the whole drug Cetuximab, only fragments of the antibody against EGFR, fused with a collagen-binding domain loaded on a collagen scaffold, can be engineered. When transplanted into an acute SCI, it can function in a similar way. The modified collagen scaffold facilitated the maturation of the newborn neurons, which differentiated from the endogenous neural stem cells. Synaptic connections detected in the lesion suggest the integration of newborn cells into the existing neuronal network [73].

The biggest limitation of the above section is the fact that the majority of experiments were performed in rodent cellular or animal models. More information on in vitro differentiation could be obtained from human iPS cells differentiated into ependymal cells to recapitulate some of the crucial experiments confirming neurogenesis described in Section 3. However, there are limited protocols describing the differentiation of iPS into ependymal cells [77]. Some studies even question the possibility of neurogenesis in the spinal cord by any means [78,79]. Ren et al. [78] performed large crush injuries across the spinal cord and found minimal SCEC migration with less than 2% contribution of SCECs to the total newly proliferated scar-forming astrocytes. Confirmation of neurogenesis in human spinal cord tissue requires a post mortem analysis of individuals with spinal cord injury compared to non-traumatic causes (controls). The study of Cawsey et al. [80] reported a significant increase in the percentage of SCECs that were nestin-positive (a marker of neural progenitor cell response) between the controls and trauma cases in human samples. Nestin-positive cells were seen in cervical, thoracic and lumbar levels of the spinal cord, suggesting that nestin reactivity is not just a localized reaction to injury. Further characterization of SCECs in the human spinal cord is, therefore, required to determine their role after injury and to confirm the character of neural progenitor cells.

### 3.2. The Reduction of Neuroinflammation

Primary injury in the spinal cord is followed by secondary injury, which is characterized by the creation of an inflammatory and inhibitory microenvironment that contains inhibitors for axon regeneration and repair, such as myelin-associated glycoproteins, reactive astrocytes, activated microglia and infiltrated macrophages [81]. These neuroinflammatory conditions inhibit axon regeneration and negatively influence the activated NSCs to differentiate into neurons [82,83]. Therefore, several treatments targeting neuroinflammation can also positively influence SCECs proliferation and/or differentiation, such as the aforementioned valproic acid. VPA exhibits neuroprotective benefits by reducing SCI-induced apoptosis, neurotoxicity, inflammation and autophagy during the secondary injury period. In addition, VPA upregulates pro-survival neurotrophic proteins, attenuating the inflammatory environment and protecting the remaining neural cells from secondary damage, as reviewed in [41]. Several anti-inflammatory strategies target activated microglia/microphages. One of the key regulators of microglial differentiation is the interaction between colony-stimulating factor 1 (CSF1) with its receptor CSF1R. Therefore, the reduction in activated CD68+ microglia/macrophages using an inhibitor of CSF1 receptor reduced inflammation and led to an increased number of neurons differentiated from SCECs [84]. Similar results can be obtained when shifting the polarization of the macrophage M1 pro-inflammatory phenotype toward the M2 pro-regenerative phenotype. The overexpression of Rictor (an important component of mTOR pathway that is responsible for axonal growth) in spinal cord injury shifted the macrophage polarization around the lesion from the M1 to the M2 phenotype and increased neurogenesis in the lesion epicenter [85].

Immunization with neural-derived peptides (INDP) shifts the inflammatory microenvironment toward a more permissive one, which is characterized by an increase in anti-inflammatory cytokines and the production of neurotrophic factors. These effects are carried out by stimulating an M2 macrophage phenotype. Moreover, a significant increase in neurogenesis, mainly at the central canal and at both the dorsal and ventral horns of INDP-treated animals, was even observed in animals in the chronic stage of SCI [86].

However, neuroinflammation has both beneficial and detrimental effects and there are several aspects that must be taken into consideration. A typical example is a methylprednisolone (MP) steroid, which is commonly used after spinal cord injury in patients for its effect on the attenuation of secondary injury. MP inhibits the activation and proliferation of various inflammatory cell types in animal models of SCI, by reducing the production of inflammatory cytokines/chemokines and free radicals, as well as inhibiting lipid peroxidation. However, it was shown that the application of MP inhibits the proliferation and migration of SCECs and oligodendrocytes after an SCI, not only in rodents but also in nonhuman primates [87].

### 3.3. Neuromodulation with Physical Factors

Neuromodulation is necessary for the participation of NSCs in neural repair [88]. Researchers recently found that physical factors, such as electric, magnetic and ultrasound effects, can stimulate the activation of stem cells in the CNS [89,90]. A robust increase in NSC proliferation in the adult mouse intact brain was reported after a 2-week application of repeated transcranial magnetic stimulation (rTMS) at both low (1 Hz) and high (30 Hz) frequencies [91]. These experiments were repeated in vitro with similar results, showing that the application of rTMS for 1 week with both 1 and 30 Hz also facilitated NSC proliferation and neuronal differentiation [91]. Furthermore, the very-low-frequency electromagnetic field could activate the excitability of neural progenitor cells and regulate T-type calcium channels, both of which are connected with electrical activity and have the potential formation of neural circuits [92,93]. To date, there is no evidence regarding the ability of rTMS to mobilize SCECs after an SCI. SCEC mobilization was achieved by extracorporeal shock waves, which were applied 4 weeks after an SCI in rats. In the treated animals, increased proliferation of SCECs was detected in the ependymal layer of the central canal and the injured posterior horn. Some limited differentiation into neuronal and glial phenotypes was also reported [94]. Physical factors can be relatively easily translated to human medicine since they are non-invasive and can serve in the future as part of rehabilitation or supportive treatment in patients with spinal cord injury.

### 3.4. In Vivo Reprogramming

Finally, a new approach regarding how to increase neurogenesis in the injured spinal cord emerged in the last few years with new advances in the reprogramming field. In vivo reprogramming techniques have the potential to convert non-neuronal cells into neurons via the forced expression of transcription factors. Recently, there have been several studies showing that the overexpression of different pro-neural transcription factors can convert endogenous glial cells into neurons (reviewed in [95]). The most often utilized TF is Sox2, a neural progenitor marker that keeps the balance between stem cell renewal and differentiation. The overexpression of Sox2 in astrocytes in the injured spinal cord resulted in the conversion into doublecortin positive neuroblasts. These cells can mature into neurons and connect with endogenous motoneurons. In combination with VPA administration, these cells can survive for 210 days, and the yield of neurons is increased twofold [53]. Interestingly, reprogramming with Sox2 does not skip the proliferating phase of neuroblasts; therefore, one astrocyte can give rise to several neurons. The Sox2 strategy was used not only for reprogramming astrocytes but also NG2 glia. Another advantage appears to be the fact that reprogrammed neurons are not only glutamatergic but also GABAergic [96]. NeuroD1 is another important TF that is needed for neuronal differentiation. The overexpression in astrocytes converts the astrocytes into glutamatergic neurons in a 1:1 ratio. As a pro-survival TF, it also reduces apoptosis in newly generated neurons [97]. Genes can be delivered using different viral vectors. Gene delivery with adeno-associated viruses (AAVs) have several advantages over lentiviruses or retroviruses. Transduction with AAVs does not require proliferating cells; therefore, AAVs can transduce astrocytes after the inflammation peak is over and the acute lesion is already closed. The use of AAVs is therefore suitable for the chronic stage of a SCI. It is easier to reprogram astrocytes in gray matter than in white matter, which is most likely due to neurotrophic support from the endogenous surrounding host neurons [98]. Recently, the type-II-clustered, regularly interspaced, short palindromic repeat and the Cas9 nuclease (CRISPR/Cas9) system from bacteria was utilized for genome editing. The overexpression of the TFs Islet-1 (Isl1), together with Ngn2, can convert astrocytes in the spinal cord gray matter into functional motoneurons [99]. These cells express motoneuron markers, such as CHAT and HB9, and were able to fire action potentials and project their axons into the sciatic nerve to innervate muscles. This technique was used in healthy spinal cords; how effectively these neurons will integrate into host neural circuits or replace damaged motoneurons after spinal cord injury and improve functional outcome needs to be investigated.

In vivo reprogramming has made major progress; however, there are several questions that remain unanswered. The cell population around the lesion is very heterogenous and using different reprogramming factors may not generate identical subtypes of neurons. Moreover, induced neurons may not have the developmental clues for axon guidance to make precise axonal projections within the injured tissue. Worse still, the induced neurons may disrupt host neural circuits or form abnormal ones.

## 4. Conclusions

The translation of methodology, resulting in the replacement of the depleted population of neurons after spinal cord injury, is an important issue in regenerative medicine. Recent science is currently not able to solve whether neurogenesis in the spinal cord can serve as a source of replacement neurons in human medicine. Some studies completely deny neurogenesis in the spinal cord [78,79]. Most likely, the type and severity of the spinal cord lesion, as well as the microenvironment, play a role in the induction of neurogenesis. Further studies highlight the fact that, in the human adult spinal cord, the spinal canal is not as prominent as in rodents and the remaining ependymal cells do not proliferate; therefore, they cannot serve as a pool for cell replacement [100]. Conversely, other researchers describe the increase in nestin-positive cells in the human spinal cord after injury [80]. In any event, most likely only subtle and advanced techniques that aim at an optimal interventional strategy focused on combined strategies can lead to the eventual success in promoting neurogenesis and/or reconstructing the damaged neuronal circuits and improving functional outcomes. Manipulating the intrinsic properties of ependymal cells in the central canal or gene editing the converting glial cells into neurons together with changing the nonpermissive environment could be the future of personalized medicine for patients with spinal cord injury.
